# Supplementary material for: Implementation of a sample design for a survey of program participants using time-location sampling
Source: PLoS One. 2023 May 17;18(5):e0285877. doi: 10.1371/journal.pone.0285877 (PMC10191336; doi:10.1371/journal.pone.0285877)
Supplement: S1 Fig — (DOCX) [file pone.0285877.s001.docx]

S1 Fig. Overview of WIC ITFPS-2 interviews administered to subsamples

|  |  | **Subsample** | |
| --- | --- | --- | --- |
| **Interview (months)** |  | **Core** | **Supplemental** |
|  | **Prenatal** | ✓ |  |
|  | **1** | ✓ | ✓ |
|  | **3** | ✓ | ✓ |
|  | **5** | ✓ |  |
|  | **7** | ✓ | ✓ |
|  | **9** | ✓ |  |
|  | **11** | ✓ |  |
|  | **13** | ✓ | ✓ |
|  | **15** | ✓ |  |
|  | **18** | ✓ |  |
|  | **24** | ✓ | ✓ |
|  | **30** | ✓ | ✓ |
|  | **36** | ✓ | ✓ |
|  | **42** | ✓ | ✓ |
|  | **48** | ✓ | ✓ |
|  | **54** | ✓ | ✓ |
|  | **60** | ✓ | ✓ |
|  | **72** | ✓ | ✓ |
