# Supplementary material for: Implementation of a sample design for a survey of program participants using time-location sampling
Source: PLoS One. 2023 May 17;18(5):e0285877. doi: 10.1371/journal.pone.0285877 (PMC10191336; doi:10.1371/journal.pone.0285877)
Supplement: Attachment — Submitted filename: Response to Reviewers.docx [file pone.0285877.s003.docx]

February 16, 2022

Manuscript Number: PONE-D-21-33776

Original Title: “Designing a sample of program participants using time-location sampling”

Revised Title: “Implementation of a sample design for a survey of program participants using time-location sampling”

Dear Dr. Zalm,

On behalf of my co-authors, I would like to thank you for consideration of our manuscript for publication in PLOS ONE. We appreciate the reviewers’ careful review of our manuscript and their insightful comments. We have revised the manuscript to address the comments raised by the reviewers, and are now submitting the revised manuscript for your consideration.

Sincerely,

Yumiko Siegfried

ATTACHMENT. Authors’ Responses to Reviewers’ Comments

Journal Requirements:

When submitting your revision, we need you to address these additional requirements.

1. Please ensure that your manuscript meets PLOS ONE's style requirements, including those for file naming. The PLOS ONE style templates can be found at

<https://journals.plos.org/plosone/s/file?id=wjVg/PLOSOne_formatting_sample_main_body.pdf> and

[https://journals.plos.org/plosone/s/file?id=ba62/PLOSOne_formatting_sample_title_authors_affiliations.pdf](https://secure-web.cisco.com/1y0ns0OITBNEs3lw8bLCTBmgd3orgq8u7hwKQj6-m05YZrRmtBJFRHXk6MavBFQCEe5yRK3IRBFSQOBiw8CAxjVGfm-BNj0qr4UYUPHhgi0QiIvSRpmK_CKE9DeQnOSyYIuy8R4As5T9kATxze3a42bpbXYY9obKtiwu6C9F_bxdNUUne7I5sJod9Wmv5GphSta5xWAQucwWCdfNCWqV8ix48cLUE-gcp9zEu3QxGFEDSJLkpAlFEavIPktDQ7DLb/https%3A%2F%2Fjournals.plos.org%2Fplosone%2Fs%2Ffile%3Fid%3Dba62%2FPLOSOne_formatting_sample_title_authors_affiliations.pdf)

*We have reformatted the manuscript in accordance with PLOS ONE’s style requirements.*

**Review Comments to the Author**

**Reviewer #1:** I had the pleasure of reviewing this manuscript and recommend that it be accepted without major revisions. The authors clearly stated the need for this manuscript, detailed their method by describing an example application, and provided a reasonable discussion. Aside from minor stylistic choices the authors might revisit before publication, depending on the journal's style (e.g., using "data" in the singular - line 167, or capitalizing State Agencies while keeping local agencies in lower case), I have no recommendations for these authors. Excellent work.

*We have revised line 167 (in the original version) as suggested. We also made related grammatical changes.*

*We have revised the manuscript so that state agencies and local agencies are both in lower case.*

**Reviewer #2:** See attached file. [pasted below]

**Reviewer #3:** The manuscript “Designing a sample of program participants using time-location sampling” details the approach developed to use time-location sampling (TLS) in 2013 to enroll a cohort for the WIC Infant and Toddler Feeding Practices Study-2. This complex sample design needed to account for numerous considerations, including identifying the appropriate types of locations, estimating flow rates, determining days/hours of operation to establish study recruitment lengths, randomizing the assignment of recruitment windows to balance different flow periods, adequate staffing, and calculating appropriate survey weights. This carefully designed time-location sample enabled a probability sample to be produced. The manuscript is well-written in easy-to-understand language and details the approach, challenges and solutions, and limitations of this study. Because TLS generally produces non-probability samples, the strategy detailed here may be helpful to others looking to implement a similar approach. To that end, I have some comments and suggestions provided below to help make the paper more generalizable.

Major Comments:

1. There are only four citations, and only one of those is directly related to TLS. I would suggest extending the literature review in Section 1, perhaps highlighting in particular other examples of where TLS was used to create a probability sample.

*As suggested, we have added additional references, including examples of where TLS was used to create a probability sample.*

2. How were the various sample sizes determined? Why 80 sites (line 177)? Why 98 women per site (line 222)? Information on how these and other sample size numbers were obtained (and why) would be helpful to other practitioners. At least provide a reference and justification if using a standard formula.

*We revised the title to “Implementation of…” rather than “Design of…”, inserted a paragraph to explain that the development of the sample design and the detailed weighting methodology are beyond the scope of this manuscript, and inserted a reference to a report containing details of the sample design and weighting.*

3. The “Computation of survey weights for analysis purposes” section needs more detail. At minimum, a formula and/or citation should be provided. What would be even more beneficial and strengthen the work is to provide some detail about how the survey weights were actually calculated since you repeatedly stress that what makes this survey unique is creating a probability sample from TLS. That makes it seem like figuring out the survey weights is non-trivial, particularly given the complexity of your design and adjustments needed to the procedure. Even if the goal of this paper is to lay out the approach and not focus on data analysis/final estimates, the approach is incomplete without knowing the weights. This would help make the work much more generalizable.

*We added some clarifications, and inserted a cross-reference to a report that contains details about the computation of the survey weights.*

4. Lines 454-458: I am not sure that the regression line is the best way to demonstrate your point here. Two things to consider – 1) You mention the slope is positive (0.53), but don’t mention if this is actually statistically significantly different than 0 (i.e, do you reject the null hypothesis H0: beta1=0?). If not, even if the slope is positive, it could still just be due to chance. 2) You mention the outlier in the next paragraph. Is the slope still as positive (and/or still significantly different than 0) with the outlier removed? I think you could still have made your point without fitting the regression model, which adds these extra complexities/assumptions (e.g. considering case-influence statistics, if it’s appropriate to actually treat the sites as independent given the strata, etc.)

*Plot and text were revised to remove the regression line and references to it.*

Minor Comments:

1. Line 148-150: It’s stated here that the sample was designed to “oversample those with certain characteristics to increase the precision of estimates for subgroups of interest.” Although these characteristics are mentioned later (if I’m interpreting correctly, they are mother’s race, ethnicity, trimester at WIC enrollment, pre-pregnancy BMI, household composition, and income), I think it would be helpful to mention them here.

*Revised as suggested.*

2. I would recommend moving Figure S2 into the main text. The descriptions in the “Sampling of WIC sites” section were complex and having the figure there would be very helpful.

*Revised as suggested.*

3. Relatedly, I’d recommend omitting figures 2 and 3, since they each provide little information since the pre and postnatal bars sum to 100% and the core and supplemental bars also sum to 100%. Those numbers/percents could be given in a table instead for easier comparison, as the exact number of live births is hard to read from the barplot.

*We removed Figs 2 and 3.*

4. The conclusion felt a little abrupt. Having a paragraph about lessons learned, or what could be done differently for a future study, would also help make the paper more generalizable.

*Revised as suggested.*

6. PLOS authors have the option to publish the peer review history of their article ([what does this mean?](https://secure-web.cisco.com/1g0ah8c59IuhaPuYde932GMBQKF4dB9Cof95_9xIE0E68X9Oj2VFk259sABITpXU9256YfgLUyVssAsNR2yoIOvtoHYUANM5o4zYRWteQPiQnMrENBYy8yKrFteO5CMrXFXmS5UwPfWznBwLpOJOCBZh3BTRuxlw4FlzoAyUwY1xQCSqtL3qByKGM8i22MWpiz6FWqSGShHR_vQ79fsMNjHT2QnADqp_PRyMc8tvFK6sPtoareT5843NzOS7ge8Cq/https%3A%2F%2Fjournals.plos.org%2Fplosone%2Fs%2Feditorial-and-peer-review-process%23loc-peer-review-history)). If published, this will include your full peer review and any attached files.


If you choose “no”, your identity will remain anonymous but your review may still be made public.


**Do you want your identity to be public for this peer review?** For information about this choice, including consent withdrawal, please see our [Privacy Policy](https://secure-web.cisco.com/180U0MU606GEUFVwuG53BjBKw08n-U6_6ZhoaEJdgzSin1mmuO-s4hIF6fAyqjUQXnD7EH0fX_qEtDRltj-UmAIHr4nCbMS0fmQwEvOD4eY1U2vmL_1Zzo0Sg3h2KYbfLzMa8VYkWfczBvKNP1Qax7BA4J8_PmQuAAfu-3XJGFVen70THne4f78VsiLJl3irVba_O_IgQ16u0F1CCHF2zhtAet4wF57OIHj8yI_6P9imFypV0ZsPl9wn3s45vvaAV/https%3A%2F%2Fwww.plos.org%2Fprivacy-policy).

Reviewer #1: No

Reviewer #2: No

Reviewer #3: No

[NOTE: If reviewer comments were submitted as an attachment file, they will be attached to this email and accessible via the submission site. Please log into your account, locate the manuscript record, and check for the action link "View Attachments". If this link does not appear, there are no attachment files.]

While revising your submission, please upload your figure files to the Preflight Analysis and Conversion Engine (PACE) digital diagnostic tool, [https://pacev2.apexcovantage.com/](https://secure-web.cisco.com/1Iv2dV8R0O7BoUUJQABdOeJN5cOh12ih6Am0Iv27omHpuh6o4FxF2dOr_eFglmuBBXkv6d9B6niQWFQ-tOlMK0NI9a2AFVbleMgWQlwLTvU_EIEUe3cukVuyp9jgQX5OD_6HGK0qs9awOXdrcf8sVusyjOz60QOCXqkFFd2wMQKVnObyCRm29AaEkJ07gz7hmWsDId8W0y6FjPWsBr8t6oZ8OGdBAhGO8j_r_3NrcCgq69A6fGVK0hQdnd1qcbgWe/https%3A%2F%2Fpacev2.apexcovantage.com%2F). PACE helps ensure that figures meet PLOS requirements. To use PACE, you must first register as a user. Registration is free. Then, login and navigate to the UPLOAD tab, where you will find detailed instructions on how to use the tool. If you encounter any issues or have any questions when using PACE, please email PLOS at [figures@plos.org](mailto:figures@plos.org). Please note that Supporting Information files do not need this step.

**Comments from Reviewer #2**

The paper describes the implementation of time‐location sampling for a longitudinal survey of infant and toddler feeding practices, with participants based on enrolments in the Special Supplemental Nutrition Program for Women Infants and Children (WIC). This presents a number of issues at different stages of the implementation of the design, and the procedures used to overcome these challenges are described.

Overall I like the idea of an article describing the implementation of a cohort survey design and illustrating the ways in which this affects the field operations and the survey data. However, I have some reservations about the paper in its current form:

1. The title of the paper is “Designing a survey…”, but in fact there is little discussion of the different choices that make up the design, and the design is essentially taken as fixed, apart from the adjustments while the survey was in the field to try to meet the required recruitment sizes. This is why I have said “implementation” in my initial description above. For example:
   - there is no mention of the target accuracy requirement and how this relates to the sample size
   - the authors mention the need for reasonable estimates for specific subpopulations, but neither describe them nor say what the targets are
   - the time‐location sampling is effectively a cluster sample, but there is no assessment of the design effect and the balance between number of clusters and number of observations in each cluster
   - line 69‐71 discusses the impact of flow rate on staffing efficiency, but not on design efficiency.

These are all the kinds of considerations I would expect in a paper that was actually about the design. I suggest a change in title to reflect what the manuscript does focus on. Even so, a description of the design is needed, but could be provided by a reference to a technical report which describes it in more detail.

*As suggested, we have changed the title from “Designing a sample of program participants using time-location sampling” to “Implementation of a sample design for a survey of program participants using time-location sampling”. We have also added references to a technical report (Borger et al. (2022)) that provides more detail on the sample design.*

2. The manuscript claims that the sample design produces a probability sample, and this is given as a key benefit, but there is no evaluation of this. It seems largely to be true at most of the design stages, but I am particularly concerned that almost two thirds of reporting units were ineligible (line 385). I agree that these are all the smallest reporting units, but this therefore forms a cut‐off design, which is not a probability design (as many units will have a zero probability of selection). In addition this is for units, and there is a further stage of removal of ineligible sites, which are nevertheless part of the target population (Fig. S2); can you estimate the proportion of eligible sites (ie sites with non‐zero selection probabilities in your design)? If the authors want to claim that this approximates a probability design, then there needs to be some evaluation, for example (at a basic level) of what proportion of the WIC population is excluded by this approach. Ideally there would be some assessment of whether the excluded units/people have different characteristics, for which some population information would be helpful, but if that is not available you could still examine whether there is a relationship between the size of the location and the characteristics of the people in WIC. The conclusion in line 476‐477 is I think true, but I don’t think it is sufficiently demonstrated in this example.

*We inserted a statement of the proportion of enrollments excluded due to the exclusion of low-flow sites. However, the exclusions do not result in undercoverage error, because the study is not designed to cover and does not purport to cover enrollments at the low-flow or geographically ineligible sites. That is, the study population is defined with these exclusions duly noted, and there are no adjustments to the survey weights to represent these exclusions.*

3. I wanted more information on *how* the weights were calculated (or equivalently, what the selection probabilities were, lines 372‐380):
   - For the pps selections this is fairly clear, but I wondered whether any reporting units were sufficiently big that they were certain to be selected?

*Yes, there were reporting units that were sufficiently large as to be selected with certainty. These units had a probability of selection equal to 1, so the overall probability of selection of a particular new enrollee was equal to their probability of selection within the reporting unit (which took into account the length of the site recruitment period relative to the study recruitment period as well as the subsampling of participants into the core and supplemental samples).*

- how do you deal with the cut‐off reporting units in calculating weights?

*As noted in our response to comment #2 above, the study is not designed to cover and does not purport to cover enrollments at the low-flow or geographically ineligible sites. Therefore, there are no adjustments to the survey weights to represent these exclusions.*

- if I understood correctly, there are 9 release groups one week apart, and all sites selected are allocated to one release group, so the selection probability at this stage (conditional on already being included in the sample) is 1. But the collection period at a given site is longer than the one week between release groups. This means that people registering in different weeks have different probabilities to be included in the sample. For example, there are 18 weeks and 5 days of the data collection period, and so a person registering for WIC in week 1 of this period can be included only if the unit they belong to is allocated to group 1, the chance of which is 5/80. But a person registering in week 3 can be included if the unit they belong to has a data collection period of 3+ weeks and the unit is selected to group 1, 2 or 3, which has probability 25/80. Or if their unit had a data collection period of only two weeks, they could be included only if the unit was selected in group 2 or 3, which has probability 20/80. And so on. All this needs explaining.

*We inserted details of the computation of the probability of assigning the particular site recruitment period within the study recruitment period.*

- I think it would help to be more explicit about the core sample and core+supplemental sample weightings.

*Our main intention of this section of the paper was to note the effect the length of the recruitment period had on the survey weights, and to provide a brief summary of what components are taken into account in creating the weights. We inserted a reference to a technical report that provides details on the computation of the survey weights.*

4. One of the main messages from your paper is about how the design had to be adjusted based on achieved recruitment rates. But I want to know what this does to the inclusion probabilities (and therefore weights), and what impact it has on the target design parameters (variances etc)

- line 363‐364: what does the adjustment of recruitments windows do the inclusion probabilities?

*The adjustments to the recruitment windows were accounted for in the computation of the weights. We inserted a parenthetic statement to that effect into the section on Implications of TLS for the Computation of Survey Weights. Because these adjustments to the recruitment windows were fixed constants across all sites, they did not affect the variation in the inclusion probabilities.*

- line 370‐371: what does the removal of subsampling in the supplemental sample do to the variances for target subgroups?

*Removal of the subsampling in the supplemental sample results in increased sample sizes for subgroups that were previously being subsampled, thereby improving the precision of estimates for target subgroups. (Although the removal of the subsampling does produce more variable inclusion probabilities within the subgroups, the effects on precision are more than offset by the relative increase in nominal sample sizes for the subgroups.)*

5. The relation to the existing literature is not sufficiently made, particularly in the introduction. I expected to see this approach compared with other implementations of time location sampling (the International Passenger Survey in the UK is one, though it is not called by this name; there are others). There are several papers comparing location sampling with other approaches, eg McKenzie & Mistiaen (2009), and you should appropriately comment on how your approach compares to these. What is it about your approach that makes it a probability sample when others aren’t (line 91)?

*We have included additional references in the Introduction, and added a paragraph at the end of the Introduction that contrasts our approach to other applications of TLS and describes why probability sampling is relatively straightforward in our application when it isn’t in some other applications.*

I also have some more detailed comments:

a. It would help to have a description of the WIC enrolment process up front; the authots clearly know the context well, but a summary to help the reader would make the paper easier to follow.

*We added information about enrollment, including the fact that at the time of recruitment for this study, participants were required to enroll in WIC in-person by visiting a WIC site.*

b. The study is a cohort study because it follows a defined group… I think you should use this rather than “longitudinal study” which is more general, or at least give it as an alternative description.

*Revised to “longitudinal cohort study”.*

c. line 114‐116: what is the waiver worth in terms of additional recruitment (%)? This would be useful information for other survey designers.

*All participants consented verbally, and signed consent forms were obtained for 85 percent of study participants. This information has been added to the manuscript.*

d. line 156: I assume “census” relates to the PC 2010; I think you need a bit more text to say what PC 2010 is and what information you can obtain from it.

*We have expanded discussion of the PC 2010.*

e. line 214‐5: How was the half decided (another design decision as in 1 above). Is it random, or structured in some way? Why half rather than 60% (say)?

*Meeting the precision requirements for the rarest subgroups required essentially double the sample size that was required for the more prevalent subgroups (those supported by the core sample). Therefore, the full sample (designed to meet the precision requirements for the rare groups) was subsampled at 50% to create the core sample. We have added text to the manuscript to clarify this point.*

f. line 230‐1: I expect some significant seasonality in births. Does this feed through to WIC enrolments? Specifically, is the January rate a good predictor of the Jul‐Nov rate?

*We used updated WIC enrollment information from July 2012 to calculate the recruitment window lengths. We have also inserted mention of the potential effect of seasonality in births.*

g. line 255: can you find a better word than “overage” which looks like someone who is too old?

*Revised to “surplus”.*

h. line 285: I couldn’t work out what “the latter” referred to. Do you just mean “This”?

*Revised as suggested.*

i. line 309‐310: What happens to parents with multiple births (twins etc)? This should be included in the description of WIC (point a).

*We inserted a sentence to describe how we handled multiple births, and included this aspect in our added description of WIC enrollment.*

j. Line 321‐322, 332‐334: Needs more information on how this worked in practice. Was the form handed over to the recruiter, who then chased the subject down the corridor? (OK, I jest… but needs more detail). Line 417‐419 suggests that subsampling took place *before* recruitment, so that’s an extra step in the process which appears to need to happen more or less immediately. How did that work?

*We have included additional details of how the subsampling occurred (as part of the screening process).*

k. line 327‐330: I agree with you that it is important to minimise the non‐identification of eligible person. But after you make this statement, the paper makes no further mention on the topic. So how did you minimise this probability in this survey?

*We have added the details as suggested.*

l. line 340: “a few characteristics above” needs to be more precise.

*Revised as suggested.*

m. line 380: Is “nonresponse” here structural nonresponse where certain parts of the sample were not sent a questionnaire for non‐key interviews? What of general nonresponse?

*Our use of the word “nonresponse” here was intended to refer to nonresponse among those eligible and designated to receive the particular interview. We’ve added text to clarify this.*

n. line 399‐401: a histogram of recruitment window lengths might provide a better summary?

*We have added a histogram of recruitment window lengths.*

o. line 431‐432: you may have achieved the main design requirement, but not the oversampling of specific populations? An objective evaluation of what was and was not achieved from the original design specification would be helpful.

*We inserted a table that gives ratios of actual vs. expected sample sizes by subgroup, with discussion of the findings.*

p. line 454‐458: I don’t find this explanation convincing. If it was just variability, then the scatter of points would be wider, but the slope of the line would be 0 because the flow predictors would be satisfactory. Because there is a slope, flows at smaller sites were generally smaller than estimated, and larger than estimated at the largest sites. But in fact a linear fit may not be best, and I particularly want to know how it is changed if you remove the one outlying point (which appears to have high leverage). An alternative interpretation might be that expected flows were generally overestimated across all sizes.

*We revised the plot to remove the regression line. Corresponding text has also been revised to remove the mention to linear regression.*

q. line 482: I understood that you gathered information on the flow rates and used those as inputs. If there is an estimation process involved, you need to describe it.

*We revised the sentence to say “Gathering enrollment count information….” rather than “Estimating flow rates…”*

r. line 486‐487: Randomising assignment to recruitment periods is good, but you neither discuss nor assess balance.

*The balancing across heavier and lighter flow periods was implicit in the randomization of assignment of the site recruitment periods. Sites with heavier-than-usual flows during their recruitment periods have excess enrollees, and sites with lighter-than-usual flows during their recruitment periods have shortfalls of enrollees. The study did not collect the enrollment data (for the portion of the study recruitment period that falls outside the site recruitment period) from the sites that would be needed to explicitly examine the balance.*

s. line 490‐491: lines 308‐310 of the manuscript explain why individuals’ frequency of visits to eligible locations is not needed for this survey (!).

*We intended for the bulleted list to be a list of potential issues and considerations that should be addressed in designing a TLS study, and that our application illuminated many of these issues. However, we can see that our original wording might have implied that our application addressed all of these issues. We have revised the text to clarify this.*

t. Figs 1, 2 and 3 take up a lot of space for not much information. A table might be better?

*We decided to convert the original Fig 1 to Table 1, and remove the original Figs 2 and 3 since relevant information is presented in the text.*

PLOS ONE asks for typos to be corrected at revision, so here are some small edits:

- line 73: replace second “of” by “to” *Revised as suggested.*
- line 170: replace “select” by “generate” *Revised as suggested.*
- line 188: insert “the” before “160” *Revised as suggested.*
- line 303: delete “a” before “child” *Revised as suggested.*
- line 389, 392 and elsewhere: “a total of” adds nothing, please remove. *We removed all occurrences of “a total of”, and “totals of”, except for when removing it would cause the sentence to start with a number (in numeric form).*
- line 408‐9: delete “In terms of the recruitment model” *Revised as suggested.*
- line 446: insert “the” before “recruitment” *Revised as suggested.*

Reference

McKenzie, D. J., & Mistiaen, J. (2009). Surveying migrant households: A comparison of census‐based, snowball and intercept point surveys. *Journal of the Royal Statistical Society: Series A* **172** 339‐360.
