# Supplementary material for: Implementation of a sample design for a survey of program participants using time-location sampling
Source: PLoS One. 2023 May 17;18(5):e0285877. doi: 10.1371/journal.pone.0285877 (PMC10191336; doi:10.1371/journal.pone.0285877)
Supplement: Attachment — Submitted filename: Response to Reviewers.docx [file pone.0285877.s004.docx]

ATTACHMENT. Authors’ Responses to Reviewers’ Comments

Thank you for the careful revision of your paper. Following the first round of review (where I was one of the referees) I have taken over as Guest Editor for your manuscript, so I am now unmasked. The revised manuscript is substantially improved over the first version. Some further changes are, however needed.

The following issues must be addressed in a revision:

1. There is an important discrepancy between the responses to the reviewers and what the revised manuscript actually says. In the response to the reviewers you say of the exclusions of the smaller and geographically remote sites:

“the exclusions do not result in undercoverage error, because the study is not designed to cover and does not purport to cover enrollments at the low-flow or geographically ineligible sites. That is, the study population is defined with these exclusions duly noted, and there are no adjustments to the survey weights to represent these exclusions.”

But in the manuscript I read:

- a representative sample of infants enrolled in WIC was needed (line 29)
- Our approach generated a probability sample (line 35-36)
- a national sample of Federal nutrition assistance program participants (line 83-84)
- our approach was designed to yield a probability sample of new enrolees (line 106-107)
- a representative sample of WIC enrollees (line 151)
- The sample was intended to represent the national population of infants enrolled in WIC for the first time either while the mother is pregnant, or postnatally before 3 months of age, whose mothers are at least 16 years old and speak either English or Spanish. (line 152-154)

These two points of view are not consistent. The manuscript needs to explain what part of the population is covered, and about which you can make representative or probability-based inferences. I don’t have a particular issue if you want to define the target population as all infants enrolled in WIC, but it needs to be clear that the sampling reaches only a smaller population, and that you must therefore assume that the characteristics of the part of the population that is not covered are the same as those of the part that is covered. This kind of approach is, after all, standard. But it needs to be clearly explained.

*In order to be explicit about the effects of the exclusions, we have made modifications to the manuscript in the places you indicated.*

2. In the response to the referees you say “Removal of the subsampling in the supplemental sample results in increased sample sizes for subgroups that were previously being subsampled, thereby improving the precision of estimates for target subgroups.” But in fact you have achieved the required overall recruitment by removing subsampling, so the target subgroups (the ones that you intended the design to oversample) are no longer oversampled (except to the extent that they were before you changed the design). It is in fact the non-target subgroups which will receive larger sample sizes. Some assessment of the impact of this must be included.

*Table 2 gives the ratios of the actual to expected enrolled sample sizes, by subgroup.*

In addition I have some further comments which you should consider in a revision:

a. line 227-8: Is the replacement unit similar in size because it was in the same stratum, or did you make them more similar in size than that? I note that with only 4 units per stratum the options for choice are rather limited (though there could be a wider choice where the unit is subsampled within a LA)

*The replacement unit was the unit, within the same stratum as the original unit, that was closest in size to the original unit. We revised the wording to be more clear about this.*

b. line 260: Insert “(or screen out)” after “recruit”

*This change was made.*

c. line 288: “are” -> “were”

*This change was made.*

d. line 436: L_h_/L might be a reasonable approximation, but, as explained in my original review, it is not strictly true because the days do not have equal probabilities of inclusion in the sample (the days at the beginning and end of the study period are less likely to be included, unless you allow periods that "wrap" around to cover the beginning and the end, which is not very practical operationally). I think you need to explain this (and note that Borger et al. (2022) uses L_h_/L too).

*Thank you. You are correct that it is not appropriate to characterize* L_h_/L as the probability of selecting the particular set of days within the study recruitment period. We have revised the wording to instead describe this factor as an estimate of the proportion of new enrollees during the study recruitment period who enrolled during the set of recruitment days at the site.

e. line 526-530: I have a different explanation/ suspicion about the estimates of flow rates, but your explanation is also possible. If it was just variability, I'd still expect a fit to be flat. But I think no further adjustment to the text is needed.
f. You response to the referees about weights says that you added a reference to a report with details. I looked at the report and it is minimally sufficient; I note that the weighting is described only in words and no equations are given. You should still consider providing additional details about the weighting process in the paper, especially how it was adapted to changes made in the field, since that is the key topic in your paper. For example, certainty units are not mentioned.

*In this revision of the manuscript, we have provided additional details on the computation of the weights.*

g. In Fig. 1 I don’t find it clear whether “160 units / 42 SAs” (and other similar) means that there are 202 things all together; I suspect not, but you could help with something like “160 units incl 42 SAs”.

*The counts of SAs provided in the figure correspond to the number of SAs associated with the units. You are correct that these are not additional units. We have revised the figure to replace “## SAs” with “(in ## SAs)”.*
