# Supplementary material for: Implementation of a sample design for a survey of program participants using time-location sampling
Source: PLoS One. 2023 May 17;18(5):e0285877. doi: 10.1371/journal.pone.0285877 (PMC10191336; doi:10.1371/journal.pone.0285877)
Supplement: Attachment — Submitted filename: PONE-D-21-33776 referee report for authors.pdf [file pone.0285877.s002.pdf]

PONE-D-21-33776 referee report for the authors

The paper describes the implementation of time-location sampling for a longitudinal survey of infant and toddler feeding practices, with participants based on enrolments in the Special Supplemental Nutrition Program for Women Infants and Children (WIC). This presents a number of issues at different stages of the implementation of the design, and the procedures used to overcome these challenges are described.

Overall I like the idea of an article describing the implementation of a cohort survey design and illustrating the ways in which this affects the field operations and the survey data. However, I have some reservations about the paper in its current form:

1. The title of the paper is “Designing a survey…”, but in fact there is little discussion of the different choices that make up the design, and the design is essentially taken as fixed, apart from the adjustments while the survey was in the field to try to meet the required recruitment sizes. This is why I have said “implementation” in my initial description above. For example:
    - there is no mention of the target accuracy requirement and how this relates to the sample size
    - the authors mention the need for reasonable estimates for specific subpopulations, but neither describe them nor say what the targets are
    - the time-location sampling is effectively a cluster sample, but there is no assessment of the design effect and the balance between number of clusters and number of observations in each cluster
    - line 69-71 discusses the impact of flow rate on staffing efficiency, but not on design efficiency.

   These are all the kinds of considerations I would expect in a paper that was actually about the design. I suggest a change in title to reflect what the manuscript does focus on. Even so, a description of the design is needed, but could be provided by a reference to a technical report which describes it in more detail.
2. The manuscript claims that the sample design produces a probability sample, and this is given as a key benefit, but there is no evaluation of this. It seems largely to be true at most of the design stages, but I am particularly concerned that almost two thirds of reporting units were ineligible (line 385). I agree that these are all the smallest reporting units, but this therefore forms a cut-off design, which is not a probability design (as many units will have a zero probability of selection). In addition this is for units, and there is a further stage of removal of ineligible sites, which are nevertheless part of the target population (Fig. S2); can you estimate the proportion of eligible sites (ie sites with non-zero selection probabilities in your design)? If the authors want to claim that this approximates a probability design, then there needs to be some evaluation, for example (at a basic level) of what proportion of the WIC population is excluded by this approach. Ideally there would be some assessment of whether the excluded units/people have different characteristics, for which some population information would be helpful, but if that is not available you could still examine whether there is a relationship between the size of the location and the characteristics of the people in WIC. The conclusion in line 476-477 is I think true, but I don’t think it is sufficiently demonstrated in this example.
3. I wanted more information on *how* the weights were calculated (or equivalently, what the selection probabilities were, lines 372-380):
    - For the pps selections this is fairly clear, but I wondered whether any reporting units were sufficiently big that they were certain to be selected?
    - how do you deal with the cut-off reporting units in calculating weights?
    - if I understood correctly, there are 9 release groups one week apart, and all sites selected are allocated to one release group, so the selection probability at this stage

(conditional on already being included in the sample) is 1. But the collection period at a given site is longer than the one week between release groups. This means that people registering in different weeks have different probabilities to be included in the sample. For example, there are 18 weeks and 5 days of the data collection period, and so a person registering for WIC in week 1 of this period can be included only if the unit they belong to is allocated to group 1, the chance of which is 5/80. But a person registering in week 3 can be included if the unit they belong to has a data collection period of 3+ weeks and the unit is selected to group 1, 2 or 3, which has probability 25/80. Or if their unit had a data collection period of only two weeks, they could be included only if the unit was selected in group 2 or 3, which has probability 20/80. And so on. All this needs explaining.

- I think it would help to be more explicit about the core sample and core+supplemental sample weightings.

4. One of the main messages from your paper is about how the design had to be adjusted based on achieved recruitment rates. But I want to know what this does to the inclusion probabilities (and therefore weights), and what impact it has on the target design parameters (variances etc)
    - line 363-364: what does the adjustment of recruitments windows do the inclusion probabilities?
    - line 370-371: what does the removal of subsampling in the supplemental sample do to the variances for target subgroups?
5. The relation to the existing literature is not sufficiently made, particularly in the introduction. I expected to see this approach compared with other implementations of time location sampling (the International Passenger Survey in the UK is one, though it is not called by this name; there are others). There are several papers comparing location sampling with other approaches, eg McKenzie & Mistiaen (2009), and you should appropriately comment on how your approach compares to these. What is it about your approach that makes it a probability sample when others aren't (line 91)?

I also have some more detailed comments:

a. It would help to have a description of the WIC enrolment process up front; the authots clearly know the context well, but a summary to help the reader would make the paper easier to follow.
b. The study is a cohort study because it follows a defined group… I think you should use this rather than "longitudinal study" which is more general, or at least give it as an alternative description.
c. line 114-116: what is the waiver worth in terms of additional recruitment (%)? This would be useful information for other survey designers.
d. line 156: I assume "census" relates to the PC 2010; I think you need a bit more text to say what PC 2010 is and what information you can obtain from it.
e. line 214-5: How was the half decided (another design decision as in 1 above). Is it random, or structured in some way? Why half rather than 60% (say)?
f. line 230-1: I expect some significant seasonality in births. Does this feed through to WIC enrolments? Specifically, is the January rate a good predictor of the Jul-Nov rate?
g. line 255: can you find a better word than "overage" which looks like someone who is too old?
h. line 285: I couldn't work out what "the latter" referred to. Do you just mean "This"?
i. line 309-310: What happens to parents with multiple births (twins etc)? This should be included in the description of WIC (point a).
j. line 321-322, 332-334: Needs more information on how this worked in practice. Was the form handed over to the recruiter, who then chased the subject down the corridor? (OK, I jest… but needs more detail). Line 417-419 suggests that subsampling took place *before* recruitment, so

that's an extra step in the process which appears to need to happen more or less immediately. How did that work?

k. line 327-330: I agree with you that it is important to minimise the non-identification of eligible person. But after you make this statement, the paper makes no further mention on the topic. So how did you minimise this probability in this survey?
l. line 340: "a few characteristics above" needs to be more precise.
m. line 380: Is "nonresponse" here structural nonresponse where certain parts of the sample were not sent a questionnaire for non-key interviews? What of general nonresponse?
n. line 399-401: a histogram of recruitment window lengths might provide a better summary?
o. line 431-432: you may have achieved the main design requirement, but not the oversampling of specific populations? An objective evaluation of what was and was not achieved from the original design specification would be helpful.
p. line 454-458: I don't find this explanation convincing. If it was just variability, then the scatter of points would be wider, but the slope of the line would be 0 because the flow predictors would be satisfactory. Because there is a slope, flows at smaller sites were generally smaller than estimated, and larger than estimated at the largest sites. But in fact a linear fit may not be best, and I particularly want to know how it is changed if you remove the one outlying point (which appears to have high leverage). An alternative interpretation might be that expected flows were generally overestimated across all sizes.
q. line 482: I understood that you gathered information on the flow rates and used those as inputs. If there is an estimation process involved, you need to describe it.
r. line 486-487: Randomising assignment to recruitment periods is good, but you neither discuss nor assess balance.
s. line 490-491: lines 308-310 of the manuscript explain why individuals' frequency of visits to eligible locations is not needed for this survey (!).
t. Figs 1, 2 and 3 take up a lot of space for not much information. A table might be better?

PLOS ONE asks for typos to be corrected at revision, so here are some small edits:

- line 73: replace second "of" by "to"
- line 170: replace "select" by "generate"
- line 188: insert "the" before "160"
- line 303: delete "a" before "child"
- line 389, 392 and elsewhere: "a total of" adds nothing, please remove.
- line 408-9: delete "In terms of the recruitment model"
- line 446: insert "the" before "recruitment"

Reference

McKenzie, D. J., & Mistiaen, J. (2009). Surveying migrant households: A comparison of census-based, snowball and intercept point surveys. *Journal of the Royal Statistical Society: Series A* **172** 339-360.
